# Two Transcripts of FBXO5 Promote Migration and Osteogenic Differentiation of Human Periodontal Ligament Mesenchymal Stem Cells

**DOI:** 10.1155/2018/7849294

**Published:** 2018-04-19

**Authors:** Lin Liu, Kun Liu, Yanzhe Yan, Zhuangzhuang Chu, Yi Tang, Chunbo Tang

**Affiliations:** ^1^Jiangsu Key Laboratory of Oral Diseases, Nanjing Medical University, Nanjing, Jiangsu, China; ^2^Department of Dental Implantology, Affiliated Hospital of Stomatology, Nanjing Medical University, Nanjing, Jiangsu, China

## Abstract

**Objectives:**

Enhanced migration and osteogenic differentiation of mesenchymal stem cells (MSCs) are beneficial for MSC-mediated periodontal tissue regeneration, a promising method for periodontitis treatment. FBXO5, a member of the F-box protein family, is involved in the osteogenic differentiation of MSCs. Here, we investigated the effect of FBXO5 on human periodontal ligament stem cells (hPDLSCs).

**Materials and Methods:**

hPDLSCs were isolated from periodontal ligament tissue. Lentivirus FBXO5 shRNA was used to silence FBXO5 expression. Two transcripts of FBXO5 were overexpressed and transduced into hPDLSCs via retroviral infection. Migration and osteogenic differentiation of hPDLSCs were evaluated using the scratch migration assay, alkaline phosphatase (ALP) activity, ALP staining, alizarin red staining, western blotting, and real-time polymerase chain reaction.

**Results:**

The expression of FBXO5 was upregulated after osteogenic induction in hPDLSCs. FBXO5 knockdown attenuated migration, inhibited ALP activity and mineralization, and decreased RUNX2, OSX, and OCN expression, while the overexpression of two transcript isoforms significantly accelerated migration, enhanced ALP activity and mineralization, and increased RUNX2, OSX, and OCN expression in hPDLSCs.

**Conclusions:**

Both isoforms of FBXO5 promoted the migration and osteogenic differentiation potential of hPDLSCs, which identified a potential target for improving periodontal tissue regeneration.

## 1. Introduction

Periodontitis, one of the most widespread oral chronic inflammatory diseases, is the main cause of adult tooth loss and may be associated with diseases such as diabetes and atherosclerotic cardiovascular diseases [1, 2]. At present, periodontitis treatment involves conventional methods such as scaling, root planning, and guided tissue regeneration; however, these methods fail to achieve the ideal periodontal tissue regeneration or control inflammation [3]. Mesenchymal stem cell- (MSC-) mediated periodontal tissue regeneration is regarded as a promising method for periodontitis treatment [4]. MSCs are multipotent cells with self-renewal capacity and multidirectional differentiation ability. In addition, MSCs are identified as reliable seed cells for tissue regeneration [3, 5–8]. However, the decreased number and impaired function of periodontal ligament stem cells (PDLSCs) in patients with periodontitis pose difficulty in achieving the desired tissue regeneration [9, 10]. The transplantation of autologous or allogeneic MSCs into periodontal lesion areas was shown to promote periodontal tissue regeneration. When exposed to an inflamed microenvironment, exogenous MSCs could promote tissue formation through their own differentiation or by activating endogenous progenitor cells [11, 12]. Human PDLSCs (hPDLSCs) possess superior abilities to differentiate into bone, cementum, and periodontal ligament and may serve as a potential source for tissue engineering [13–16]. In comparison with human bone marrow MSCs, hPDLSCs offer several advantages [17, 18]. First, hPDLSCs are easily accessible and may be collected during orthodontic procedures or from the third molar. Second, hPDLSCs display higher proliferation rate and express specific transcription factors distinct from other types of dental stem cells such as dental pulp and periapical follicular stem cells [19, 20]. Third, hPDLSCs form cementum-like and periodontal ligament-like structures in immunocompromised mice after transplantation and may be the preferred choice to meet our goal to regenerate periodontal structures [19]. These unique characteristics deem hPDLSCs as one of the best candidates for future dental clinical applications. Accelerated bone regeneration necessitates the migration of hPDLSCs to the exact area, followed by their differentiation. hPDLSCs possess osteoblastic and cementoblastic lineages to regenerate periodontal tissue and maintain periodontal ligament integrity [4]. However, during clinical practice, poor adhesion, migration, and differentiation of delivered cells limit the therapeutic efficiency when hPDLSCs are transplanted in situ to periodontal defects [21]. Therapeutic intervention should ideally aim at dampening the inflammatory immune response and regenerating the anatomical structure and function of periodontal region [22]. It is of note that, in all of the preclinical and clinical studies, the engraftment of MSCs into damaged tissues via migration to enhance tissue repair/regeneration is a crucial process for clinical efficacy [23]. The process of bone development involves migration of cells with osteogenic potential (mesenchymal stem cells) to bone defect area and subsequent differentiation into the osteogenic lineage [24]. Thus enhancing the migration and osteogenic differentiation of MSCs is beneficial for MSC-mediated periodontal tissue regeneration.

F-box protein is a component of the ubiquitin protein ligase complex called SCFs (SKP1-cullin-F-box). This complex functions in phosphorylation-dependent ubiquitination [25–27]. F-box family is involved in the regulation of MSC functions and cell differentiation [28]. In addition, the F-box protein Fbxw7 was shown to control osteogenesis and chondrogenesis by targeting OASIS and BBF2H7 [29]. Furthermore, Fbxw7 may negatively regulate osteogenesis by targeting RUNX2 by ubiquitin-mediated degradation in a GSK3*β*-dependent manner [30]. A recent study highlighted the importance of FBL12 and degradation of p57KIP2 in the regulation of osteoblast cell differentiation [31]. Both FBXL11 and FBXL10 may regulate osteo/dentinogenic differentiation in MSCs [32]. FBXO5 (F-box only protein 5; also known as early mitotic inhibitor-1 [EMI1]) is one member of the F-box protein family, which is a key cell-cycle regulator that promotes S-phase and M-phase entry by inhibiting anaphase-promoting complex/cyclosome (APC/C) activity [33]. Previous study performed microarray analysis to identify the differential coding and long noncoding transcript expression profiling between noninduced and osteogenic-differentiated human MSCs. The bioinformatic analysis of microarray data showed that the expression of FBXO5 changed a lot after osteogenic differentiation [34]. This reminded us that FBXO5 may also regulate the function of hPDLSCs.

In this study, we investigated the role of FBXO5 in the regulation of hPDLSCs functions. FBXO5 was shown to activate the migration and osteogenic differentiation of hPDLSCs. Our results identified FBXO5 for improving tissue regeneration and periodontitis treatment. This information is expected to enhance the osteogenic differentiation of MSCs for tissue regeneration applications.

## 2. Materials and Methods

### 2.1. Cell Isolation, Cultures, and Characterization

All research involving human stem cells was approved by the Ethics Committee of the Affiliated Stomatological Hospital of Nanjing Medical University. Premolars were collected from six healthy orthodontic patients (12–16-year old) under approved guidelines set by the Jiangsu provincial dental hospital with informed consent. Teeth were disinfected with 75% ethanol and then washed with phosphate-buffered saline (PBS). hPDLSCs were isolated, cultured, and identified as previously described. Briefly, primary hPDLSCs were separated from the periodontal ligament in the middle third of the root. The periodontal ligament tissue was subsequently digested in a solution of 3 mg/mL collagenase type I (Worthington Biochemical Corp., Lakewood, NJ, USA) and 4 mg/mL dispase (Roche Diagnostics Corp., Indianapolis, IN, USA) for 1 h at 37°C. Primary hPDLSCs were grown in a humidified incubator under 5% CO_2_ at 37°C in Gibco® *α*-MEM (with GlutaMAX™) supplemented with 15% fetal bovine serum (FBS; Invitrogen, Carlsbad, CA, USA), 100 U/mL penicillin, and 100 *μ*g/mL streptomycin (Invitrogen). Passaged primary hPDLSCs were cultured in MSC medium (ScienCell, San Diego, CA, USA) and the medium was changed every 3 days. The obtained hPDLSCs were characterized as previously described [21].

### 2.2. Plasmid Construction, Viral Infection, and Cell Identification after Viral Infection

The plasmids were constructed using standard methods; all structures were verified by the appropriate restriction digestion and/or sequencing. The mRNA and protein sequences of FBOX5 were searched from NCBI website. Transcript a and transcript b cDNAs from hPDLSCs fused to a hemagglutinin (HA) tag were produced using a standard polymerase chain reaction (PCR) protocol. The two sequences (HA-FBXO5 a and HA-FBXO5 b) were subcloned into the pQCXIN retroviral vector between* Not*I and* Bam*HI restriction sites. Short hairpin RNAs (shRNAs) with complementary sequences of FBXO5 were subcloned into LV3 lentiviral vector (GenePharma, Suzhou, China). The target sequences for shRNAs were as follows: FBXO5 shRNA (FBXO5sh), 5′-GCTGTCATGTATTGGGTCACC-3′; control shRNA (CONTsh), 5′-TTCTCCGAACGTGTCACGT-3′.

Viral packaging was prepared according to the manufacturer's protocol (Clontech Laboratories, Addgene). For viral infections, hPDLSCs were plated overnight and infected with retroviruses or lentiviruses at a multiplicity of infection of 20 in the presence of polybrene (6 *μ*g/mL; Sigma-Aldrich, St. Louis, MO, USA) for 12 h according to the manufacturer's instructions. After 48 h, infected cells were selected with different antibiotics. Ectopic FBXO5 overexpression and knockdown efficiency in the transduced hPDLSCs were confirmed by real-time reverse transcription PCR (RT-PCR) and western blot analysis.

### 2.3. Scratch Migration Assay

We seeded hPDLSCs in six-well plates at a density of 2.5 × 10^5^ cells/well. A scratch was made along the diameter of the well using a 200 *μ*L pipette tip (Axygen® Corning, NY, USA) once the cells reached close to 95% confluency. The scraped cells were gently removed by washing with PBS. Fresh culture medium was added and supplemented with each one of the treatment groups as described above. Images from the same view (a circle drawn on the bottom of each well was used as a reference; the view was right inside the circle) were taken at baseline (0 h) and 24 h. The scraped width measured by ImageJ 1.42q (National Institutes of Health, USA) was used to calculate the void area (VA) and the extent of wound closure was evaluated (relative width [%] = VA/baseline area) in each group [35].

### 2.4. Induction of Osteogenic Differentiation

We seeded hPDLSCs in six-well plates at a density of 2.0 × 10^5^ cells/well. When the cells reached close to 80%–90% confluency, we changed the medium with osteogenic differentiation medium containing 10% FBS, 100 *μ*M ascorbic acid (Sigma, St. Louis, USA), 2 mM *β*-glycerophosphate (Sigma, St. Louis, USA), and 10 nM dexamethasone (Sigma, St. Louis, USA). Cells were maintained with the fresh osteogenic differentiation medium every 3 days. After 5 days of induction, ALP activity was assayed with an ALP activity kit. After 2 weeks of induction, mineralization was detected with alizarin red staining [36]. All experiments were performed in triplicate.

### 2.5. Alkaline Phosphatase (ALP) Activity and Staining

We seeded hPDLSCs in 12-well plates at a density of 1.0 × 10^5^ cells/well in an osteogenic differentiation medium. After 5 days of induction, ALP activity was assayed with an ALP activity kit according to the manufacturer's protocol (Sigma-Aldrich). ALP staining was conducted 5 days after osteogenic induction using BCIP/NBT alkaline phosphatase color development kit (Beyotime Institute of Biotechnology, Shanghai, China) according to the manufacturer's protocol.

### 2.6. Alizarin Red Detection

To detect mineralization, cells were induced for 2 weeks, fixed with 70% ethanol, and stained with 2% alizarin red (Beyotime Institute of Biotechnology). To quantify the calcium mineral density, alizarin red was destained with 10% cetylpyridinium chloride (CPC) in 10 mmol/L sodium phosphate for 30 min at room temperature. Calcium concentration was determined by measuring the absorbance of the test sample at 562 nm on a multiplate reader. A standard calcium curve was prepared with calcium dilutions in the same solution. The final calcium level in each group was normalized to the total protein concentration detected in duplicate plates.

### 2.7. Western Blot Analysis

Total protein was isolated as per the manufacturer's instructions (KeyGen Biotech, Nanjing, China). Briefly, cells were lysed in the radioimmunoprecipitation assay (RIPA) lysis buffer containing a cocktail of protease and phosphatase inhibitors and 0.5 mM phenylmethyl-sulfonyl fluoride and oscillated three times. The protein concentration was determined using bicinchoninic acid (BCA) reagent (Pierce, Rockford, IL) and equal amount of protein was resolved on a 10% sodium dodecyl sulfate polyacrylamide gel electrophoresis (SDS-PAGE) gel. The separated protein bands were transferred onto polyvinylidene fluoride (PVDF; Millipore, USA) membranes. The transblotted membranes were blocked in 5% bovine serum albumin (BSA) for 2 h at room temperature and subsequently overnight incubated with the following primary antibodies at 4°C: anti-FBXO5 (ab187144, 1 : 1000, Abcam, UK), anti-RUNX2 (ab23981, 1 : 1000, Abcam, UK), anti-OSX (ab22552, 1 : 1000, Abcam, UK), anti-OPN (ab91655, 1 : 1000, Abcam, UK), anti-OCN (ab133612, 1 : 1000, Abcam, UK), and anti-GAPDH; ab181602, 1 : 10000, Abcam, UK). After three washes in TBST for 30 min, the membranes were incubated with the secondary antibody (ab6721, 1 : 10000, Abcam, UK) for 1 h, followed by four washes with TBST for 40 min. The antibody-antigen reaction was detected using western blotting imaging system (GE Healthcare). The semiquantitative measurements were performed using ImageJ.

### 2.8. Quantitative Real-Time RT-PCR

Total RNA was extracted from hPDLSCs in each group by adding TRIzol reagent (Invitrogen, Carlsbad) to cell samples following the manufacturer's instructions. The mRNA was subjected to reverse transcription by a PrimeScript RT Master Mix kit (TaKaRa, Dalian, China). Real-time RT-PCR was performed using SYBR Premix Ex Taq kit (TaKaRa Bio, Otsu, Japan) on an ABI 7300 real-time PCR system. All primers were synthesized by the same manufacturer (Sangon Biotech, China). Real-time RT-PCR reaction conditions were as follows: 95°C for 30 s, followed by 40 cycles of 95°C for 5 s and 60°C for 31 s. GAPDH was used as an internal control gene for the standardization of mRNA levels. Relative gene expression values were calculated by the 2^−ΔΔCt^ method. PCR experiments were performed in triplicate and data were expressed as means ± standard deviation (SD). Primer sequences are shown in Table 1.

### 2.9. Statistics

All statistical calculations were performed using Statistical Product and Service Solutions (version 22.0; SPSS, Inc., Chicago, IL, USA). Student's *t*-test or one-way analysis of variance (ANOVA) was used to determine the statistical significance. A value of *P* ≤ 0.05 was considered significant.

## 3. Results

### 3.1. The Expression of FBXO5 Was Upregulated in hPDLSCs after Osteogenic Induction

**Figure 1:**
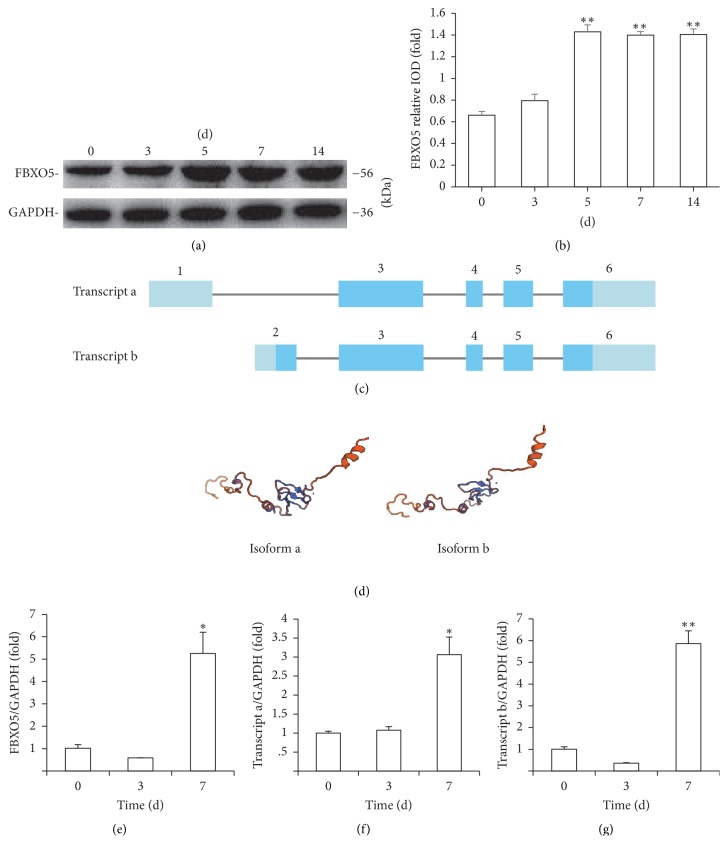
The expression of FBXO5 was upregulated after osteogenic induction in hPDLSCs. (a) Western blot results revealed that the expression of FBXO5 in hPDLSCs increased at 5, 7, and 14 days after induction. (b) Quantitative analysis of FBXO5 expression in hPDLSCs based on the results of western blotting. (c) The difference between FBXO5 transcript a (NM_001142522.2) and transcript b (NM_012177.4) was located in exons 1 and 2. (d) Comparison of the three-dimensional structure of the two isoforms using SWISS-MODEL analysis. In comparison with isoform b, isoform a had a shorter N-terminus. Real-time RT-PCR results showed that the expression of FBXO5 gene (e), transcript a (f), and transcript b (g) increased at 7 days after osteogenic induction. GAPDH was used as an internal control. Student's *t*-test was performed to determine statistical significance. Error bars represent the standard deviation (*n* = 3). ^*∗*^*P* ≤ 0.05. ^*∗∗*^*P* ≤ 0.01.

To confirm the initial expression of FBXO5 during osteogenic differentiation, we performed western blot analysis and examined the level of FBXO5 protein expression at 0, 3, 5, 7, and 14 days after osteogenic induction. We found that FBXO5 expression increased in hPDLSCs at 5, 7, and 14 days after osteogenic induction (Figures 1(a) and 1(b)). Then we found that alternative splicing of FBXO5 resulted in two transcripts: transcripts a and b. The difference between transcript a (NM_001142522.2) and transcript b (NM_012177.4) was located in exons 1 and 2 (Figure 1(c)). The comparison of the three-dimensional structure of the two isoforms with SWISS-MODEL analysis showed that transcript a had a shorter N-terminus as compared with transcript b (Figure 1(d)).

For the detection of the relative mRNA levels of two transcripts, we designed two primer pairs to specifically target the two transcripts and used real-time RT-PCR to analyze the mRNA levels in hPDLSCs at 0, 3, and 7 days after osteogenic induction. We found that the expression of both transcripts a and b increased at 7 days after osteogenic induction (Figures 1(e)–1(g)).

### 3.2. Knockdown of FBXO5 Attenuated the Migration and Osteogenic Differentiation of hPDLSCs

**Figure 2:**
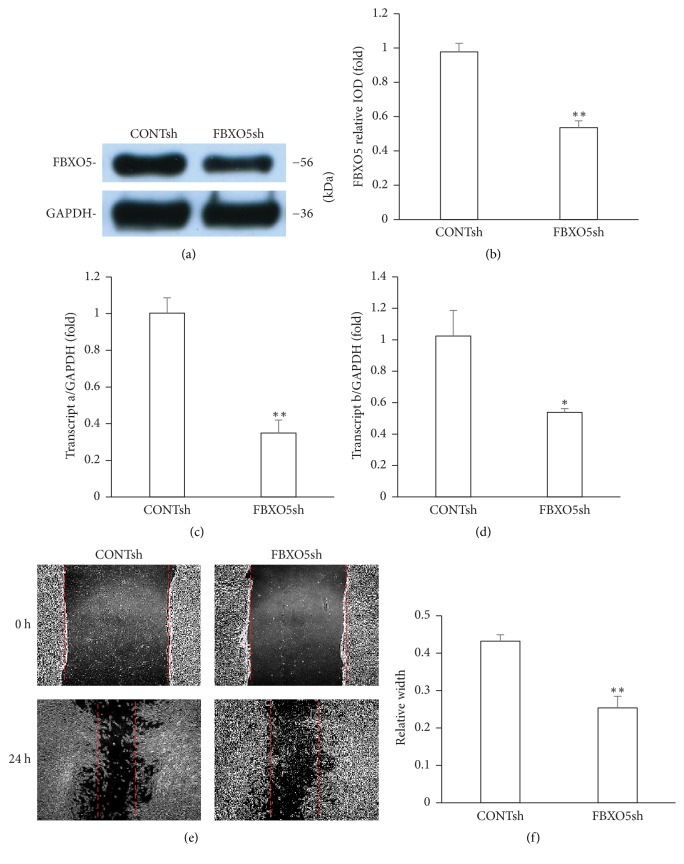
Knockdown of FBXO5 attenuated the migration of hPDLSCs. (a, b, c, d) hPDLSCs were infected with FBOX5 shRNA. Western blot (a) and quantitative analysis (b) results revealed the decreased expression of FBXO5 after the depletion of FBXO5. Real-time RT-PCR results showed the decrease in the expression of transcript a (c) and transcript b (d) after FBXO5 depletion. Scratch migration assay (e) and quantitative analysis (f) results revealed that FBXO5sh group showed a significant decrease in the rate of wound closure as compared with CONTsh group. GAPDH was used as an internal control. Student's *t*-test was performed to determine statistical significance. Error bars represent the standard deviation (*n* = 3). ^*∗*^*P* ≤ 0.05. ^*∗∗*^*P* ≤ 0.01. Scale bars represent 200 *μ*m.

**Figure 3:**
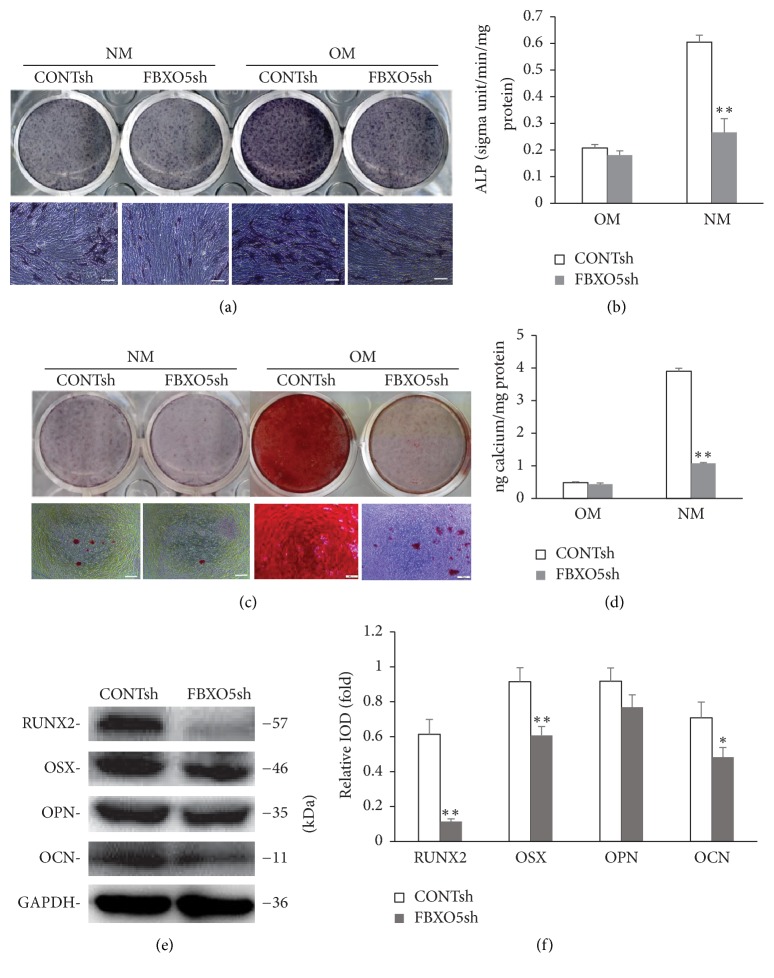
Knockdown of FBXO5 inhibited the osteogenic differentiation of hPDLSCs. Transduced cells were cultured with normal medium (NM) or osteogenic differentiation medium (OM). After 5 days of induction, alkaline phosphatase (ALP) staining (a) and ALP activity (b) results showed that ALP activity was inhibited in FBXO5sh-treated hPDLSCs. After 2 weeks of induction, alizarin red staining (c) and calcium quantitative analysis (d) results revealed that mineralization was inhibited in FBXO5sh-treated hPDLSCs. After 7 days of induction, western blot results (e) and quantitative analysis (f) revealed a significant decrease in the expression of RUNX2, OSX, and OCN in FBXO5sh group as compared with CONTsh group. GAPDH was used as an internal control. Student's *t*-test was performed to determine statistical significance. Error bars represent the standard deviation (*n* = 3). ^*∗*^*P* ≤ 0.05. ^*∗∗*^*P* ≤ 0.01. Scale bars represent 200 *μ*m.

An shRNA targeting FBXO5 was designed and introduced into hPDLSCs via lentiviral infection. After selection, the knockdown efficiency was confirmed by western blot (Figures 2(a) and 2(b)). Real-time RT-PCR results revealed a decrease in the mRNA expression of both transcripts a and b (Figures 2(c) and 2(d)). The scratch migration assay and quantitative analysis results showed a decrease in cell migration in cells depleted with FBXO5 as compared with the control group at 24 h (Figures 2(e) and 2(f)). To investigate the effect of FBXO5 on osteogenic differentiation, transduced hPDLSCs were cultured in normal medium (NM) and osteogenic differentiation medium (OM). The result showed that the osteogenic differentiation of hPDLSCs is very weak with normal medium, and there is no significant difference between FBXO5 knockdown group and control group. However, FBXO5 depletion significantly reduced ALP activity at 5 days after osteogenic induction as compared with the control group (Figures 3(a) and 3(b)). After osteogenic induction for 2 weeks, mineralization was markedly inhibited in cells treated with FBXO5sh as compared with the control group (Figures 3(c) and 3(d)), as evident from alizarin red staining and quantitative calcium measurements. In addition, western blot analysis showed that the depletion of FBXO5 significantly decreased the expression of RUNX2, OSX, and OCN at 7 days after osteogenic induction as compared with the control group; however, no significant change was observed in the expression of OPN (Figures 3(e) and 3(f)).

### 3.3. Overexpression of FBXO5 Enhanced the Osteogenic Differentiation and Migration of hPDLSCs

**Figure 4:**
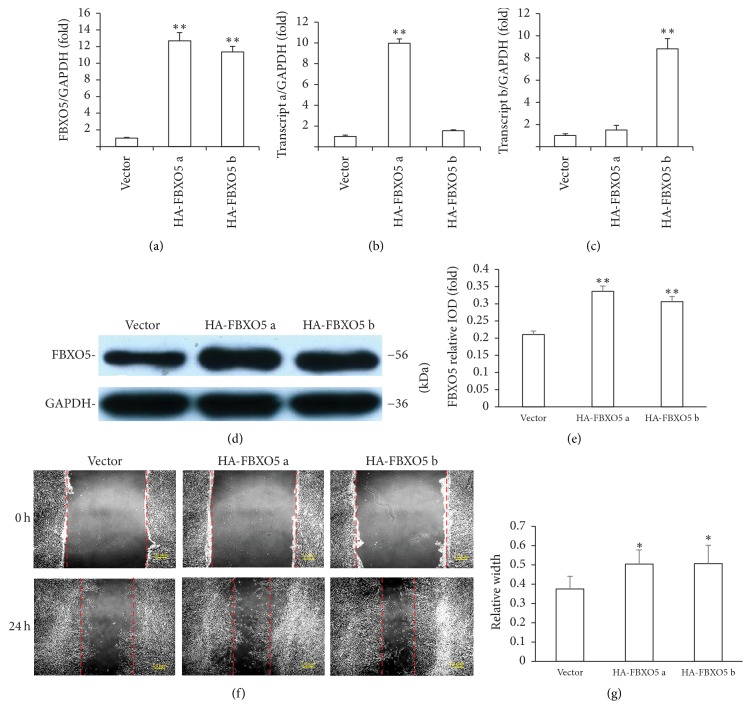
Overexpression of FBXO5 enhanced the migration of hPDLSCs. (a, b, c, d, e) HA-FBOX5 a and HA-FBXO5 b sequences were inserted into a retroviral vector and transduced into hPDLSCs via retroviral infection. Real-time RT-PCR results showed an increase in the expression of FBXO5 gene (a), transcript a (b), and transcript b (c) after FBXO5 overexpression. Western blot results (d) and quantitative analysis (e) showed an increase in the protein expression of FBXO5 after FBXO5 overexpression. Scratch migration assay (f) and quantitative analysis (g) results revealed that both HA-FBOX5 a and HA-FBXO5 b showed a significant increase in the rate of wound closure as compared with the vector group. GAPDH was used as an internal control. Student's *t*-test was performed to determine statistical significance. Error bars represent the standard deviation (*n* = 3). ^**∗**^*P* ≤ 0.05. ^**∗∗**^*P* ≤ 0.01. Scale bars represent 200 *μ*m.

**Figure 5:**
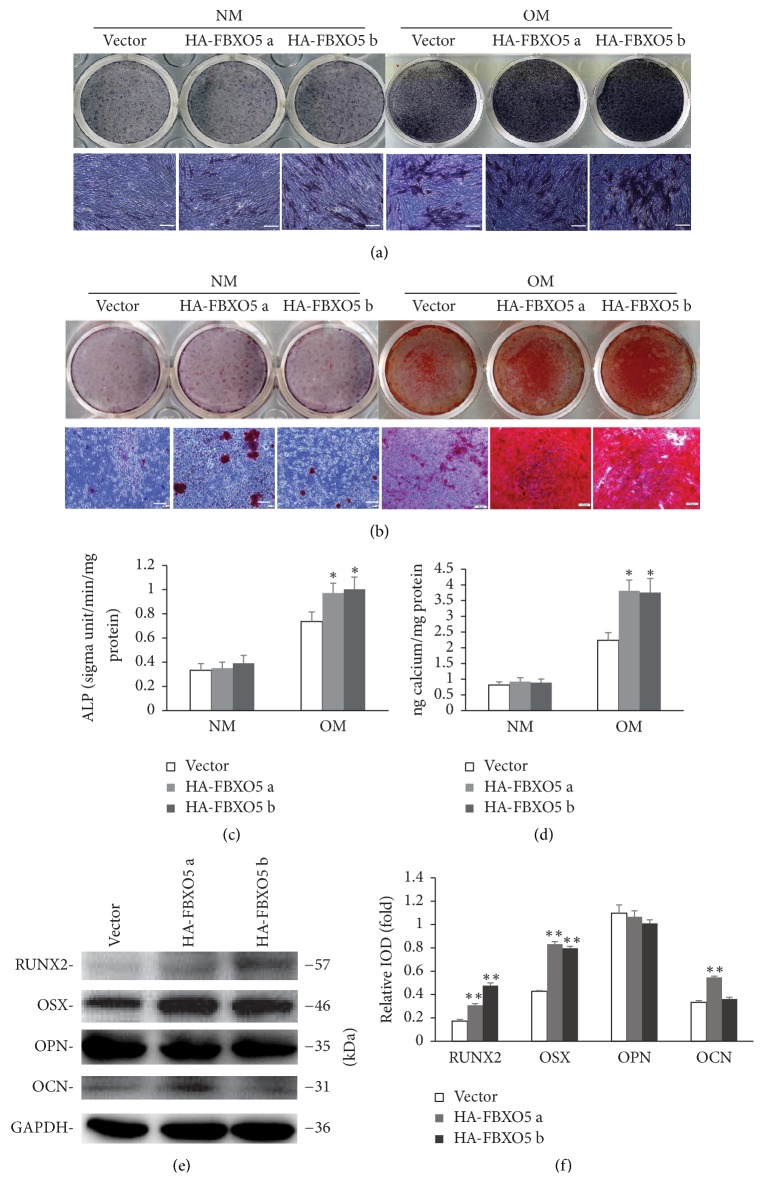
Overexpression of FBXO5 enhanced the osteogenic differentiation of hPDLSCs. Transduced cells were cultured with normal medium (NM) or osteogenic differentiation medium (OM). After 5 days of induction, alkaline phosphatase (ALP) staining (a) and ALP activity (c) results showed that both HA-FBOX5 a and HA-FBXO5 b enhanced ALP activity in hPDLSCs. After 2 weeks of induction, alizarin red staining (b) and calcium quantitative analysis (d) results revealed that both HA-FBOX5 a and HA-FBXO5 b enhanced mineralization in hPDLSCs. After 7 days of induction, western blot results (e) and quantitative analysis (f) showed a significant increase in the expression of RUNX2 and OSX in HA-FBOX5 a and HA-FBXO5 b group as compared to the vector group. The expression of OCN significantly increased in HA-FBOX5 a group compared with the vector group. GAPDH was used as an internal control. Student's *t*-test was performed to determine statistical significance. Error bars represent the standard deviation (*n* = 3). ^**∗**^*P* ≤ 0.05. ^**∗∗**^*P* ≤ 0.01. Scale bars represent 200 *μ*m.

Alternately spliced transcript variants encoding multiple isoforms have been observed for FBXO5. Whether transcripts a and b function independently or exhibit any relationship is, however, unclear. To investigate the function of these two isoforms in hPDLSCs, we inserted HA-FBOX5 a and HA-FBXO5 b sequences into a retroviral vector and transduced it into hPDLSCs via retroviral infection. After selection, the overexpression efficiency was confirmed by western blot and real-time RT-PCR analyses (Figures 4(a)–4(e)). An increase in cell migration was observed in the presence of both HA-FBOX5 a and HA-FBXO5 b as compared with the control group at 24 h (Figures 4(f) and 4(g)), as evident from the scratch migration assay and quantitative analysis results. Similarly, there is no significant difference between FBXO5 overexpressed group and control group in osteogenic differentiation when cultured in normal medium. But after osteogenic induction for 5 days, ALP activity increased in cells treated with HA-FBOX5 a and HA-FBXO5 b as compared with the control group (Figures 5(a) and 5(c)). A marked increase in mineralization was observed for the groups overexpressing HA-FBOX5 a or HA-FBXO5 b as compared with the control group, as determined by alizarin red staining and quantitative calcium measurements (Figures 5(b) and 5(d)). The results of western blot analysis showed that both isoforms a and b significantly increased the expression of RUNX2 and OSX at 7 days after osteogenic induction in hPDLSCs as compared with the control group. HA-FBXO5 a significantly increased the expression of OCN, while OCN protein level was unaffected in cells treated with HA-FBXO5 b. In addition, the expression of OPN was unaffected by HA-FBOX5 a or HA-FBXO5 b (Figures 5(e) and 5(f)).

## 4. Discussion

F-box proteins are known to be involved in the osteogenic differentiation of MSCs [26, 28–31]; whether FBXO5 affects osteogenic differentiation of hPDLSCs is, however, unclear. In present study, we found that FBXO5 promoted osteogenic differentiation and migration in hPDLSCs, suggestive of its potential role in periodontal tissue regeneration. In vitro, FBXO5 expression increased during osteogenic induction of hPDLSCs, which indicates that FBXO5 may play an important role in hPDLSCs. What is more, knockdown of FBXO5 led to a reduction of the migration and osteogenic differentiation functions in hPDLSCs. By contrast, overexpression of FBXO5 enhanced the migration and osteogenic differentiation potential of hPDLSCs. These findings suggest that FBXO5 plays a positive role in regulating the migration and osteogenic differentiation of hPDLSCs.

It is rarely reported that FBXO5 directly plays a role in cell migration. However, FBXO5 (also known as early mitotic inhibitor-1 [EMI1]) is a key cell-cycle regulator that promotes S-phase and M-phase entry by inhibiting anaphase-promoting complex/cyclosome (APC/C) activity [33, 37]. S-phase kinase associated protein 2 (Skp2) is an F-box protein that was originally discovered as a protein associated with the S-phase kinase Cdk2/cyclin A and hence its name. Skp2 protein availability is dependent on both the gene expression from Skp2 and the rate of protein degradation. Skp2 degradation is mediated by the ubiquitin ligase APC/CDH1 (anaphase-promoting complex/cyclosome and its activator CDH1) during G1 phase; EMI1 is an inhibitor of APC/CDH1 [38]. To sum up, FBXO5 might upregulate the expression of Skp2 via inhibiting the degradation of Skp2 by APC/CDH1 [39].

Previous report found that downregulation of Skp2 inhibits the viability, proliferation, colony formation, migration, and invasion and induces apoptosis of human gastric cancer cells. Chen et al. also found that Skp2-RNAi inhibits the migratory ability of SW620 cells [40]. The influences of Skp2 on cell migration and invasion are mainly mediated through its downstream factors, p27 and p21, by different mechanisms [41, 42]. Both p27 and p21 participate in regulating cell migration and adherence by regulating RhoA activity [42]. Particularly, p27 modulates these effects through interacting with RhoA to facilitate depolymerization of microfilaments [41] or with stathmin to inhibit instability of microtubules [41]. It has been shown that p27 is targeted for degradation by Skp2 as the specific substrate-recognizing and rate-limiting subunit [43]. Previous report showed that upregulation of p27 by the depletion of Skp2 led to the reduction of cell migration [44]. Based on these evidences, we speculate that FBXO5 might upregulate the expression of Skp2 and thereby decrease the expression of p27 and p21 and then promote cell migration of hPDLSCs. However, further studies are required to confirm this possibility.

Concerning the mechanism for FBXO5 promoting osteogenic differentiation of hPDLSCs, from the western blot analysis, we found that FBXO5 knockdown decreased the expression of RUNX2 and OSX, while overexpression of two transcript isoforms significantly increased the expression of RUNX2 and OSX in hPDLSCs. Several studies have highlighted the role of RUNX2 and OSX in osteoblast function at the molecular level [45]. RUNX2 and OSX are crucial and exhibit consistent strong expression at early stages of tooth development and bone formation. The coexpression of RUNX2 and OSX may enhance the downstream transcription of osteogenic genes [45, 46]. These discoveries indicated that FBXO5 may enhance the osteogenic differentiation of hPDLSCs via regulating the expression of RUNX2 and OSX. However, further investigation will be carried out to elucidate the speculation and precise molecular mechanism.

Alternative splicing allows a single gene to produce two or more similar but distinct mature mRNA variants that expand the coding capabilities of eukaryotic genomes [47, 48]. Alternative splicing is a type of posttranscriptional gene regulation and may generate several unique transcripts from a single gene-coding region, thereby resulting in multiple protein variants. This process is critical for diverse cellular processes and development [49, 50]. Alternately spliced transcript variants encoding multiple isoforms have also been observed for FBXO5 gene; we named these as isoforms a and b. Whether these two isoforms perform same functions is questionable. Functional studies with real-time RT-PCR revealed that the expression of both transcripts a and b increased at 7 days after osteogenic induction. We investigated the functions of two isoforms in hPDLSCs and found that both isoforms a and b promoted cell migration and osteogenic differentiation in hPDLSCs. However, from the western blot analysis, we found that FBXO5 isoform a, but not isoform b, significantly increased the expression of OCN. OCN is the most abundant noncollagenous protein in bone. It is considered a late marker of bone differentiation [51]. FBXO5 is one of the known human F-box proteins that serve as specificity factors for a family of ubiquitin ligases that are involved in targeting proteins for degradation across the ubiquitin proteasome system [52]. Alternative splicing of FBXO5 resulted in two transcripts: transcripts a and b. The difference between transcript a and transcript b was located in exons 1 and 2. As most alternative exons are localized on the protein surface, alternative splicing can regulate the binding to other proteins. Exons can encode complete interaction domains or part of binding domains, which changes the interaction with other proteins [53]. So we speculate that, compared to transcript a, transcript b might interact with OCN through its substrate interacting domain (such as WD repeats), and OCN is subsequently degraded by transcript b via ubiquitin-mediated proteasome degradation. However, further investigation will be carried out to elucidate the precise molecular mechanism.

## 5. Conclusion

In conclusion, our results demonstrated that FBXO5 promotes the migration and osteogenic differentiation of hPDLSCs. Both isoforms may promote osteogenic differentiation of hPDLSCs but differ in their specific regulation mechanisms. These findings highlight the potential role and clinical applications of FBXO5 in periodontal tissue regeneration. Future studies should investigate the molecular mechanism underlying FBXO5 effects on hPDLSCs.

## Figures and Tables

**Table 1:** Primer sequences used in this study.

Gene	Primer pairs	Primer sequence (5′~3′)	GeneBank accession number
FBXO5	Sense	cgctgtaattcacctgcaaa	NM_001142522.2 NM_012177.4
	Antisense	gaggagcttgccatctgaac	
FBXO5 Transcript a	Sense	gggctgagattaggacttgc	NM_001142522.2
	Antisense	agtccggaatgaacatggtt	
FBXO5 Transcript b	Sense	gctggcgccttttaagagat	NM_012177.4
	Antisense	cacttcattttgacagaaagggt	
GAPDH	Sense	gcaccgtcaaggctgagaac	NG_007073
	Antisense	tggtgaagacgccagtgga	
